# The fate of the contralateral limb after major lower limb amputation for peripheral arterial disease or diabetes mellitus

**DOI:** 10.1093/bjsopen/zrag134

**Published:** 2026-08-19

**Authors:** Robert J Leatherby, Bilal Azhar, Arsalan Wafi, Luis Ribeiro, Peter Holt, Iain Roy

**Affiliations:** St George’s Vascular Institute, St George’s University Hospital NHS Foundation Trust, London SW17 0QT, UK; Cardiovascular & Genetics Research Institute, City St George’s University of London, London, UK; St George’s Vascular Institute, St George’s University Hospital NHS Foundation Trust, London SW17 0QT, UK; Cardiovascular & Genetics Research Institute, City St George’s University of London, London, UK; St George’s Vascular Institute, St George’s University Hospital NHS Foundation Trust, London SW17 0QT, UK; Cardiovascular & Genetics Research Institute, City St George’s University of London, London, UK; St George’s Vascular Institute, St George’s University Hospital NHS Foundation Trust, London SW17 0QT, UK; St George’s Vascular Institute, St George’s University Hospital NHS Foundation Trust, London SW17 0QT, UK; Cardiovascular & Genetics Research Institute, City St George’s University of London, London, UK; St George’s Vascular Institute, St George’s University Hospital NHS Foundation Trust, London SW17 0QT, UK; Cardiovascular & Genetics Research Institute, City St George’s University of London, London, UK

## Abstract

**Background:**

The contralateral limb is at risk after major lower limb amputation (MLLA) for peripheral arterial disease or diabetes mellitus due to the systemic nature of these diseases, with a subsequent bilateral MLLA required in over 10% of patients. The present study aims to determine the natural history of the contralateral limb after MLLA for peripheral arterial disease or diabetes.

**Methods:**

A single-centre retrospective cohort study of patients undergoing MLLA for peripheral arterial disease or diabetes between January 2013 and July 2023 was performed. The primary outcome was clinically relevant contralateral limb disease (CR-CLD), defined as symptomatic disease of the contralateral limb or asymptomatic disease requiring prophylactic intervention. Statistical analysis used both survival analysis and competing risk modelling techniques.

**Results:**

A total of 531 patients were included, with 175 (33%) going on to develop CR-CLD. The leading aetiology was chronic limb-threatening ischaemia, with 79.7% of these patients presenting with tissue loss. A total of 67% of CR-CLD presented by 1 year and 81% by 2 years after index MLLA. A diagnosis of diabetes, chronic peripheral arterial disease, ischaemic heart disease, and previous contralateral vascular intervention were identified as independent risk factors for the development of CR-CLD on multivariable analysis. A total of 72.6% of patients with CR-CLD underwent intervention, 28.6% of these requiring contralateral MLLA. For those not undergoing intervention, 56.3% were turned down due to co-morbidity or frailty.

**Conclusion:**

CR-CLD after MLLA is common, and presents early after index MLLA but with late-stage clinical disease. High-risk groups are identifiable. Outcomes are poor, with many patients undergoing a contralateral MLLA or being palliated.

## Teaser

The present study investigates the fate of the contralateral limb after major amputation for peripheral arterial disease or diabetes mellitus. The authors demonstrate that clinical disease occurs in the contralateral limb of a third of patients, with 81% presenting within 2 years, and the majority with late-stage clinical disease. Outcomes in those who develop contralateral clinical disease are poor, with 29% requiring bilateral major amputation and 18% turned down for intervention.

## Introduction

Due to the systemic nature of peripheral arterial disease (PAD) and diabetes mellitus, both lower limbs are at risk of complications. For those patients with unilateral chronic limb-threatening ischaemia (CLTI), 34.5–36.4% of patients develop contralateral CLTI within 2–4 years of initial diagnosis^1,2^. These patients also appear to present later in the disease process with higher rates of tissue loss and advanced wound, ischaemia, and foot infection grading compared with unilateral patients^1,3^. For those patients undergoing major lower limb amputation (MLLA) for PAD, the risk of requiring a contralateral MLLA is 5.7% at 1 year, increasing to 11.4–11.5% in the longer term^4,5^. There is evidence to suggest this figure may be higher in the population living with diabetes, with a study inclusive of all amputations at or proximal to the transmetatarsal level showing a contralateral amputation incidence of 17 per 100 amputee-years for those with diabetes compared with 12 for those without^6^. Patients with bilateral MLLA have a very poor prognosis, with few mobilizing on a prosthesis, a poorer quality of life compared with their unilateral MLLA counterparts, and a 5-year mortality rate of 69%^7–9^, highlighting the importance of contralateral limb salvage. Contralateral limb disease and its prevention have recently been highlighted as a core outcome after MLLA and a priority for future research^10,11^.

Prevention of contralateral disease starts with medical secondary prevention. Unfortunately, this appears to be an often-missed opportunity, with only 39% of patients after MLLA being prescribed an antiplatelet and a statin, and 32% being prescribed neither in a single centre study^12^ performed in 2006. This missed opportunity for secondary prevention is particularly important as revascularization of the contralateral limb has been shown to be less effective, with lower limb bypasses demonstrating increased complication rates, graft occlusion, and MLLA for those with a contralateral MLLA compared with their counterparts without amputations^13^. Many of these patients would now undergo endovascular first revascularization based upon current evidence for high-risk patient groups^14,15^. Whether patients undergoing endovascular intervention for contralateral limb revascularization also have worse outcomes is unknown.

Based upon this, the global vascular guidelines^16^ on the management of CLTI advise at least annual follow-up for post-MLLA patients to monitor contralateral disease progression, optimize medial therapy, and manage risk factors. This recommendation is strong but only based upon weak evidence. There is little evidence to demonstrate that this recommendation is incorporated into clinical practice.

The present study establishes the fate of the contralateral limb after MLLA in a contemporary series from a metropolitan regional hospital. The incidence, time-to-event, and course of contralateral limb disease are quantified alongside identifying risk factors for disease development.

## Methods

### Study design

A single-centre retrospective cohort study was undertaken within a large regional metropolitan vascular unit. Patients who underwent a first unilateral MLLA at the centre between 1 January 2013 and I July 2023 for PAD or diabetes were included. MLLA was defined as any definitive amputation above the level of the ankle. Those patients undergoing amputation for trauma, malignancy, or congenital deformity were excluded. Patients undergoing bilateral MLLA or those who had already had a contralateral MLLA before the start of the study period were also excluded.

The present study was completed in line with the STROBE statement checklist for observational studies^17^. It was reviewed and approved by the St George’s University of London ethics and integrity department who deemed this work to be a service evaluation with no further ethical opinion required, and was conducted in accord with the ethical standards of the Committee on Human Experimentation of the institution in which the experiments were done and in accord with the ethical standards of the Helsinki Declaration of 1975.

### Outcomes

The primary outcome for the present study was ‘clinically relevant contralateral limb disease’ (CR-CLD). This was defined as symptomatic disease secondary to PAD or diabetes, or asymptomatic disease requiring prophylactic intervention, for example threshold popliteal aneurysms, occurring in the contralateral limb concurrent to, or after, index MLLA.

Other outcomes of interest for the present study were the requirement for contralateral intervention, including revascularization and amputation, and amputation-free survival after contralateral limb revascularization.

### Data sources and extraction

Data were collected from local electronic records, which are linked to regional spoke site and GP records via London Care Record and national NHS spine records for death outcomes. Demographic, co-morbidity, perioperative, morbidity, death, and contralateral limb outcome data were collected for each patient. Radiology was directly interpreted using duplex reports or diagrams and computed tomography reports alongside direct image interpretation to evaluate for significant radiological disease as a predictive variable for CR-CLD. This was defined as occlusion or flow limiting (=/> 75%) stenosis affecting in-line arterial supply to the foot.

### Statistical analysis

Data analysis was performed using ‘R’ statistical software^18^. Data were checked for normality visually using both histograms and quantile–quantile plots. Subgroup comparisons were bivariable and the χ^2^ or Fisher’s exact test was used for categorical variables and the *t*-test or Mann–Whitney *U* test for continuous variables. Univariable and multivariable modelling was performed using the ‘finalfit’ package^19^. Death was identified as a competing risk for the development of CR-CLD and therefore competing risk methodology in the form of a cause-specific Cox-proportional hazards model^20^ was used, giving hazard ratios (HRs) with 95% confidence intervals (c.i.) and *P* values. Variables for inclusion in the models were chosen based on clinical relevance and after data visualization. Collinear variables were identified both visually and using the variance inflation factor test and eliminated. Multiple imputation by chained equation (MICE) was used to account for missing data, using the ‘mice’ package^21^.

Contralateral limb ‘survival’ analysis was performed using the ‘survival’ and ‘ggsurvfit’ packages^22,23^. Time-to-event analysis with censoring was performed to produce cause-specific Kaplan–Meier ‘survival’ plots and median ‘survival’ times for the development of CR-CLD. Cumulative incidence analysis for the composite outcome of amputation-free survival was performed using the same packages to assess the role of contralateral disease severity at presentation on the outcomes of revascularization.

## Results

**Figure 1:**
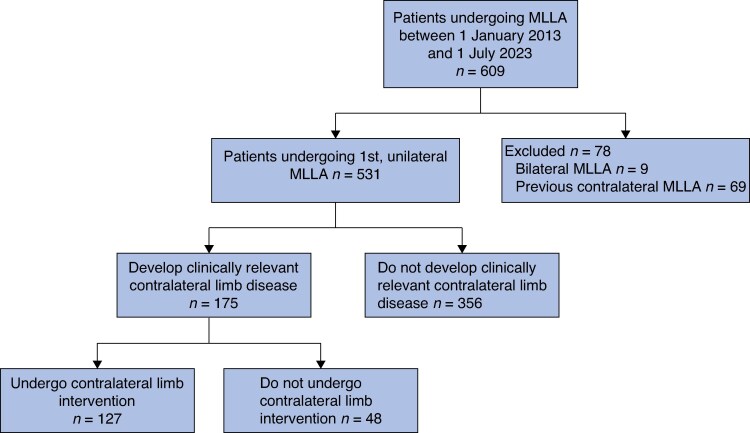
Flowchart of patients in study cohort MLLA, major lower limb amputation.

During the study period, 531 patients underwent unilateral MLLA who had not previously had a contralateral MLLA. A flow chart visualizing the cohort is presented in *Fig. 1*. The mean age of the cohort was 68.4 years, with 75.0% of patients being men. Full demographics, patient characteristics, and surgical details are presented in *Table 1*. The median survival for the cohort, irrespective of the fate of the contralateral limb, was 3.8 years (95% c.i. 3.2 to 4.6) after the index MLLA.

**Table 1:** Demographic, patient characteristics, surgical details and outcomes for the patient cohort

	Full cohort (*n* = 531)	No CL disease (*n* = 356 (67.0%))	CL disease (*n* = 175 (33.0%))	P*
Age (years), mean(s.d.)	68.4(11.8)	68.2(12.3)	68.8(11.2)	0.570
**Sex**				0.177
Male	398 (75.0%)	260 (73.0%)	138 (78.9%)	
Female	133 (25.0%)	96 (27.0%)	37 (21.1%)	
**Ethnicity**				0.075
White	242 (45.6%)	167 (46.9%)	75 (42.9%)	
Black	43 (8.1%)	22 (6.2%)	21 (12.0%)	
Asian	23 (4.3%)	13 (3.7%)	10 (5.7%)	
Other/unknown	223 (42.0%)	154 (43.3%)	69 (39.4%)	
**Smoking**				0.294
Never	255 (48.0%)	178 (50.0%)	77 (44.0%)	
Ex	151 (28.4%)	94 (26.4%)	57 (32.6%)	
Current	125 (23.5%)	84 (23.6%)	41 (23.4%)	
Hypertension	323 (60.8%)	205 (57.6%)	118 (67.4%)	0.037
Ischaemic heart disease	191 (36.0%)	105 (29.5%)	86 (49.1%)	< 0.001
Heart failure	78 (14.7%)	51 (14.3%)	27 (15.4%)	0.836
DM	305 (57.4%)	187 (52.5%)	118 (67.4%)	0.002
Atrial fibrillation	102 (19.2%)	71 (19.9%)	31 (17.7%)	0.620
Hyperlipidaemia	157 (29.6%)	91 (25.6%)	66 (37.7%)	0.005
PAD	353 (66.5%)	212 (59.6%)	141 (80.6%)	< 0.001
Chronic kidney disease	154 (29.0%)	94 (26.4%)	60 (34.3%)	0.075
**End-stage renal disease**				0.279
Dialysis	56 (10.5%)	34 (9.6%)	22 (12.6%)	
Transplant	3 (0.5%)	3 (0.8%)	0 (0.0%)	
**Cerebrovascular disease**				0.824
TIA	16 (3.0%)	11 (3.1%)	5 (2.9%)	
Stroke	72 (13.6%)	46 (12.9%)	26 (14.9%)	
Cancer	54 (10.2%)	33 (9.3%)	21 (12.0%)	0.409
**Prescribed best medical therapy**† **after index MLLA**				0.106
Yes	369 (69.5%)	233 (65.4%)	136 (77.7%)	
No	63 (11.9%)	47 (13.2%)	16 (9.1%)	
(Missing)	99 (18.6%)	76 (21.3%)	23 (13.1%)	
**Level of index MLLA**				0.171
BKA	259 (48.8%)	177 (49.7%)	82 (46.9%)	
TKA	19 (3.6%)	9 (2.5%)	10 (5.7%)	
AKA	253 (47.6%)	170 (47.8%)	83 (47.4%)	
**Primary aetiology of index MLLA**				0.244
ALI	93 (17.5%)	69 (19.1%)	24 (13.7%)	
Acute on chronic	60 (11.3%)	42 (11.8%)	18 (10.3%)	
CLTI	240 (45.2%)	149 (41.9%)	91 (52.0%)	
DFI	131 (24.7%)	91 (25.6%)	40 (22.9%)	
Other	8 (1.5%)	6 (1.7%)	2 (1.1%)	
**CL in-line flow at time of index MLLA**				0.015
No	102 (19.2%)	56 (15.7%)	46 (26.3%)	
Diseased	106 (20.0%)	51 (14.3%)	55 (31.4%)	
Yes	134 (25.2%)	89 (25.0%)	45 (25.7%)	
(Missing)	189 (35.6%)	160 (44.9%)	29 (16.6%)	

Values are *n* (%) unless otherwise stated. *The χ^2^ or Fisher’s exact test was used for categorical variables and the *t*-test or Mann–Whitney *U* test for continuous variables. †Best medical therapy defined as statin, plus antiplatelet or anticoagulant. CL, contralateral; s.d. standard deviation; DM, diabetes mellitus; PAD, peripheral arterial disease; TIA, transient ischaemic attack; MLLA, major lower limb amputation; BKA, below-knee amputation; TKA, through-knee amputation; AKA, above-knee amputation; ALI, acute limb ischaemia; CLTI, chronic limb-threatening ischaemia; DFI, diabetic foot infection.

The primary indications for these 531 patients’ index MLLA were acute limb ischaemia (ALI) in 93 (17.5%), acute-on-chronic limb ischaemia in 60 (11.3%), CLTI in 240 (45.2%), diabetic foot infection (DFI) in 131 (24.7%), and other indications for 8 (1.5%). After index amputation 79.8% of patients were discharged on an antiplatelet or anticoagulation drug, 72.9% on a statin, and 69.5% on both, which were defined as best medical therapy (BMT) for the purposes of the present study.

### Presentation of clinically relevant contralateral disease

**Figure 2:**
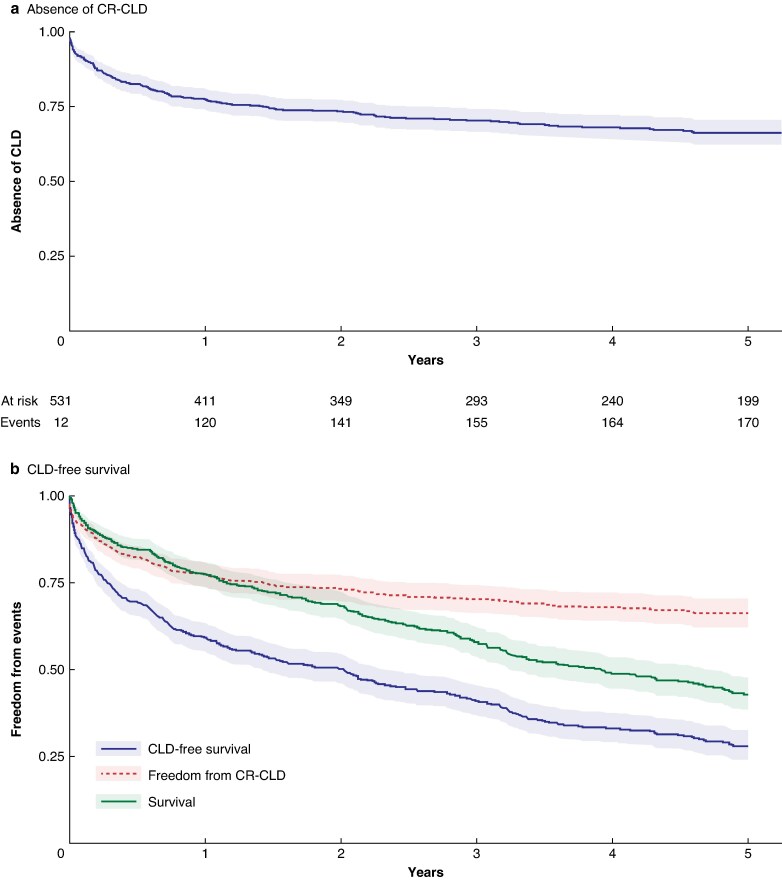
Kaplan–Meier plots of absence of CR-CLD and CLD-free survival **a** Absence of CR-CLD, **b** CLD-free survival. CR-CLD, clinically relevant contralateral limb disease.

A total of 175 patients (33.0%) went on to develop CR-CLD (*Fig. 2a*). These patients had their initial MLLA for similar indications as the full cohort (*P* = 0.244); see *Table 2* for details. These patients developed CR-CLD early after their index MLLA, with 39% presenting within 90 days, 67% presenting within 1 year, and 81% presenting within 2 years of their index amputation. Death in the follow-up period was identified as a competing risk for the development of CR-CLD. Median contralateral limb disease-free survival was 2.1 years (95% c.i. 1.7 to 2.6) for the cohort from the time of index MLLA (*Fig. 2b*).

**Table 2:** Aetiology of index MLLA and contralateral disease presentations

Primary aetiology	Index MLLA		Clinically relevant disease process in contralateral limb (*n* = 175)
	Whole cohort (*n* = 531)	Those who develop CR-CLD (*n* = 175)	
ALI	93 (17.5%)	24 (13.7%)	5 (2.9%)
A on CLI	60 (11.3%)	18 (10.3%)	3 (1.7%)
CLTI	240 (45.2%)	91 (52%)	123 (70.3%)
DFI	131 (24.7%)	40 (22.3%)	23 (13.1%)
Other	8 (1.5%)	2 (1.1%)	13 (7.4%)
Claudication	-	-	8 (4.6%)

Values are *n* (%) unless otherwise stated. MLLA, major lower limb amputation; CR-CLD, clinically relevant contralateral limb disease; ALI, acute limb ischaemia; A on CLI, acute-on-chronic limb ischaemia; CLTI, chronic limb-threatening ischaemia; DFI, diabetic foot infection; -, not available.

**Figure 3:**
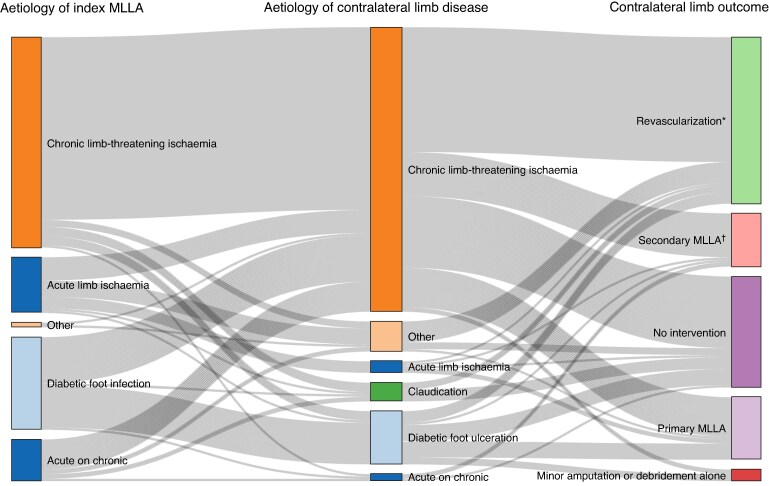
Sankey plot of aetiology of index MLLA, aetiology of contralateral disease, and contralateral limb outcomes for patients developing CR-CLD *Revascularization group includes patient who required synchronous or subsequent minor amputation or debridement. †Secondary MLLA is defined as MLLA after an attempt at revascularization. MLLA, major lower limb amputation; CR-CLD, clinically relevant contralateral limb disease.

For those 175 patients presenting with CR-CLD, 5 (2.9%) presented with ALI in their contralateral limb, 3 (1.7%) with acute-on-chronic limb ischaemia, 123 (70.3%) with CLTI, 23 (13.1%) with diabetic foot ulceration (with or without infection), 8 (4.6%) with claudication, and 13 (7.4%) with other pathology (7 popliteal aneurysms, 4 radiologically threatened stents or bypasses, 1 Charcot deformity, and 1 pseudoaneurysm). This is shown visually as a Sankey diagram in *Fig. 3*.

Of those with CLTI, 24 (19.5%) had rest pain alone (Rutherford 4) with 98 (79.7%) having tissue loss (Rutherford 5/6) at time of presentation (1 patient’s Rutherford score was unknown).

### Competing risk models

There were complete data for all predictive variables except for radiological disease of the contralateral limb, with 35.6% missing data in this field. Of the 189 patients with no contralateral imaging, 50 had no arterial imaging at all, 137 had unilateral duplex imaging of the index limb alone, and 2 had unilateral digital subtraction angiography of the index limb alone.

Cause-specific Cox proportional hazards modelling for determining aetiology was performed, with univariable and multivariable model outcomes presented in *Table 3*. Four variables were statistically significant in the final multivariable model. A diagnosis of diabetes mellitus at the time of index MLLA conferred an HR of 1.73 (95% c.i. 1.22 to 2.45; *P* = 0.002), chronic PAD at the time of index MLLA an HR of 1.91 (1.24 to 2.90; *P* = 0.003), ischaemic heart disease (IHD) HR 1.43 (1.04 to 1.98; *P* = 0.030) and previous contralateral limb vascular intervention (either revascularization or minor amputation) HR of 1.44 (1.02 to 2.04; *P* = 0.041). Contralateral limb in-line arterial flow was identified as an additional protective factor on univariable analysis (HR 0.62, 0.42 to 0.93; *P* = 0.029), but this failed to retain significance in the multivariable model (*P* = 0.241). The full model is shown in *Table 3*.

**Table 3:** Competing risk model (disease-specific Cox analysis) assessing determinants of risk for the development of clinically relevant contralateral limb disease

	Univariable HR	P*	Multivariable HR	P*
Age (1 year)	1.00 (0.99, 1.01)	0.701	0.99 (0.98, 1.01)	0.459
Male sex	1.30 (0.91, 1.87)	0.156	1.21 (0.84, 1.76)	0.310
Ex-smoker	1.32 (0.94, 1.86)	0.116	1.07 (0.74, 1.53)	0.729
Current smoker	1.08 (0.74, 1.58)	0.695	1.01 (0.68, 1.51)	0.957
Diabetes	1.67 (1.22, 2.30)	0.002	1.73 (1.22, 2.45)	0.002
Chronic PAD	2.38 (1.64, 3.47)	< 0.001	1.91 (1.25, 2.90)	0.003
Ischaemic heart disease	1.84 (1.37, 2.48)	< 0.001	1.43 (1.04, 1.98)	0.030
Heart failure	1.04 (0.69, 1.57)	0.855	0.88 (0.58, 1.35)	0.559
Dialysis dependence	1.30 (0.83, 2.03)	0.251	0.98 (0.61, 1.58)	0.938
Amputation above transtibial level	1.10 (0.82, 1.49)	0.517	1.24 (0.89, 1.71)	0.200
Previous contralateral limb vascular intervention	1.98 (1.43, 2.75)	< 0.001	1.44 (1.02, 2.04)	0.041
In-line arterial flow to foot	0.62 (0.42–0.93)	0.029	0.77 (0.51–1.17)	0.241

Values in parentheses are 95% confidence intervals. HR, hazard ratio; PAD, peripheral arterial disease. *These results are from a disease specific cox-proportional hazards model analysis.

A sensitivity analysis, removing contralateral limb in-line arterial flow from the model due to a high degree of missing data, identified the same four variables as significant on multivariable analysis.

### Contralateral limb interventions and outcomes

Contralateral limb intervention was performed in 127 (72.6%) of the 175 patients who went on to develop CR-CLD, shown in the Sankey diagram in *Fig. 3*. Of those patients undergoing intervention, 75 (59.1%) required a single intervention, and 52 (40.9%) required multiple interventions, with a mean of 1.88 interventions per patient (range 1–8). Of those patients undergoing contralateral intervention, 17 (13.4%) underwent open revascularization, 84 (66.1%) underwent endovascular revascularization, 3 (2.4%) required a hybrid procedure, 29 (22.9%) required minor (below ankle) amputation or foot wound debridement, and 50 (39.4%) required a contralateral MLLA. Contralateral MLLA was therefore required in 28.6% of patients presenting with CR-CLD, and 9.4% of the entire cohort undergoing an initial unilateral MLLA. The median survival after contralateral MLLA was 3.1 years (95% c.i. 1.8 to 5.4).

**Figure 4:**
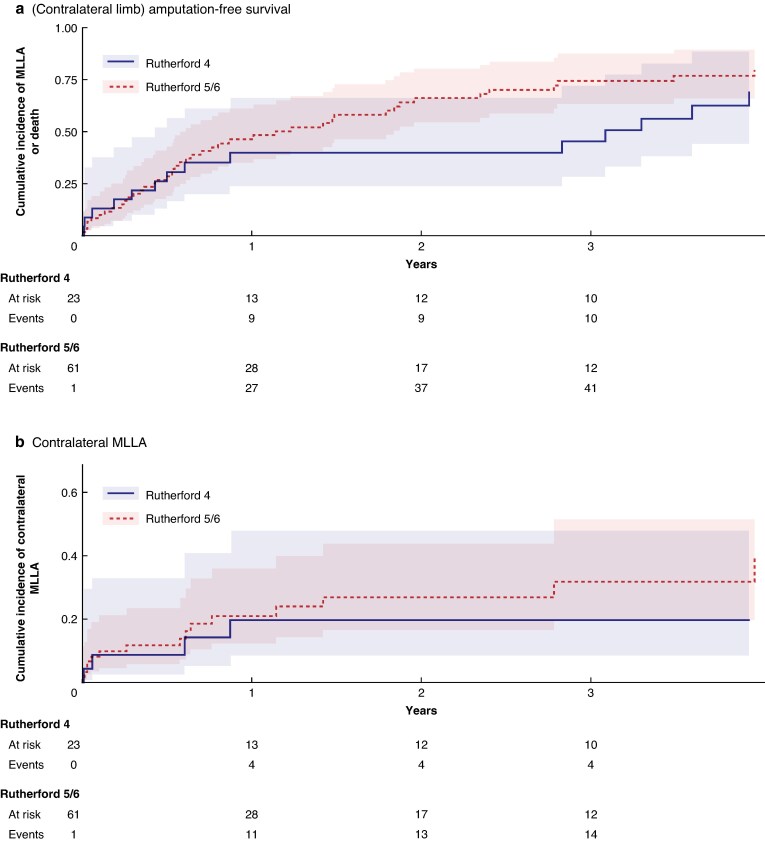
Cumulative incidence plots of (contralateral limb) amputation-free survival and contralateral MLLA by clinical severity of CLTI at time of contralateral limb revascularization **a** (Contralateral limb) amputation-free survival, **b** contralateral MLLA. MLLA, major lower-limb amputation; CLTI, chronic limb-threatening ischaemia.

A subgroup analysis by Rutherford score was performed for patients undergoing revascularization for CR-CLD due to CLTI. The two subgroups compared were those patients without tissue loss (Rutherford 4) and those with tissue loss (Rutherford 5/6). The outcome of interest was (contralateral limb) amputation-free survival. In the Rutherford 4 subgroup, 4 patients went on to have a contralateral MLLA and 11 died in the follow-up period, compared with 15 contralateral MLLAs and 28 deaths in the Rutherford 5/6 subgroup. Median time to contralateral MLLA or death was 3.1 years in those treated for rest pain compared with 1.1 years in those treated for tissue loss; however, this difference failed to reach statistical significance. Cumulative incidence plots of subgroup analysis by contralateral MLLA and amputation-free survival are presented in *Fig. 4*.

For those 48 patients who developed CR-CLD who did not go on to have intervention, 17 (35.4%) responded to medical or conservative measures alone, 4 (8.3%) had no revascularization option available, and 27 (56.3%) were turned down for intervention based upon co-morbidity or frailty, with 12 of these being moved onto an acute in-hospital or ‘fast-track’ palliative pathway.

## Discussion

The present study demonstrates that clinically relevant contralateral limb disease is common after MLLA for PAD or diabetes, with a third of patients going on to develop this. This is comparable to previous studies investigating contralateral CLTI development after a unilateral diagnosis^1,2^. The findings of the present study determined that CR-CLD presents early after a patient’s index MLLA, with 67% having presented by 1 year, and 81% by 2 years. Thirty-nine per cent of patients developed contralateral disease within 3 months of their index amputation, suggesting there is an opportunity to identify these patients before discharge or at their first follow-up appointment. Patients predominantly present with CLTI in their contralateral limb, with a much-reduced incidence of acute ischaemic presentations when comparing with the reasons for their index MLLA. This may be due to more effective treatment for ALI with anticoagulation, or a higher mortality rate in this patient group, precluding the development of contralateral disease. Patients present clinically late in their contralateral limb disease process, with 4 of 5 CLTI patients already having tissue loss at time of presentation. This again is in keeping with previous contralateral disease studies in CLTI patients^1^. These poor outcomes are despite a relatively good prescription of BMT at 69.5% on discharge after index MLLA compared with historic figures of 39%^12^.

Another key finding of the present study is the identification of risk factors that increase this risk of developing contralateral disease. The univariable competing-risk analysis identified five factors that increase risk: a diagnosis of diabetes mellitus, chronic PAD, IHD, previous contralateral limb interventions, and radiological evidence of arterial disease at time of index MLLA. All except for radiological disease remained significant on multivariable analysis, suggesting they are independent risk factors. Whilst none of these are unexpected risk factors, it does help stratify which patients are at particular risk and may benefit from enhanced clinical surveillance, as well as encouraging contralateral limb arterial imaging at time of initial MLLA. The similar HR and 95% c.i. for both diabetes and PAD highlight the importance of both conditions and their interplay in the development of contralateral disease. With podiatry commissioning mainly limited to patients with diabetes mellitus in the UK, this adds to the argument for widening access to include those with PAD.

The present study demonstrates the natural history for these patients with CR-CLD. Some 72.6% of patients went on to have contralateral limb intervention, with 40.9% of these going on to have multiple interventions. The overall risk of contralateral MLLA after unilateral MLLA is 9.4%, a similar figure to previous studies^4,5^, and this rises to 28.6% for those who develop clinical contralateral disease. The median survival rate for these patients at 3.1 years after second MLLA is comparable to unilateral amputation, but this likely reflects the more stringent patient selection criteria applied when embarking on intervention in this cohort rather than palliative care. For those patients who do not receive intervention for their contralateral disease, a majority are turned down for radiological or clinical factors and placed on a palliative pathway, with only a minority (35.4%) of these patients actively improving on medical management.

One important limitation of the present study is its retrospective nature. Whilst a trend towards better contralateral limb amputation-free survival was shown from intervention at a lower Rutherford grade, this failed to reach statistical significance due to small subgroup cohorts. However, there is good evidence to show revascularization outcomes are better for CLTI in earlier clinical disease in the general population, and there is no reason to suspect it would not apply to people with amputations^24,25^. Another limitation was that of missing data regarding radiological assessment of the contralateral limb, necessitating data imputation for variable inclusion. Given the retrospective data set, it was not possible to determine the reason for not imaging the other leg, with the possibility of skewed data missingness affecting the result of the modelling. A final limitation was the standard single appointment follow-up and hub-and-spoke set-up of the vascular service, which was likely insufficient to identify all patients developing CR-CLD, and therefore the present study may be an underestimate of the burden of disease in the community.

The present study would suggest that contralateral disease after MLLA is common, presents early after index MLLA with late-stage clinical disease, and is predictable. This adds evidence to the case for clinical contralateral limb surveillance after MLLA, as suggested by the global guidelines for CLTI^16^. If resources are limited, then this surveillance should be concentrated on the first 2 years after MLLA and can be risk-stratified to focus on these identified high-risk groups. This clinical surveillance does not necessarily need to be surgeon-led, and in fact there is good evidence for podiatry-led services in this area^26^. This is a strong case for a prospective trial in this area, evaluating the role of enhanced clinical surveillance aiming to deliver more timely intervention against the current standard of care, to see whether outcomes can be improved for this patient cohort with a high risk of bilateral MLLA or death.

Contralateral disease after MLLA is common and presents early after index MLLA with late-stage clinical disease. It is more common in those with diabetes mellitus, chronic PAD, IHD, those with multiple previous contralateral limb interventions, and those with radiological arterial disease at time of index MLLA. There is therefore a strong case for prospective assessment of the role for clinical surveillance of the contralateral limb after MLLA for PAD or diabetes mellitus.

## Data Availability

Data supporting the findings of the present study are available from the corresponding author upon reasonable request.
